# A Comparison of the ATP Generating Pathways Used by *S*. Typhimurium to Fuel Replication within Human and Murine Macrophage and Epithelial Cell Lines

**DOI:** 10.1371/journal.pone.0150687

**Published:** 2016-03-01

**Authors:** Enriqueta Garcia-Gutierrez, Amanda C. Chidlaw, Gwenaelle Le Gall, Steven D. Bowden, Karsten Tedin, David J. Kelly, Arthur Thompson

**Affiliations:** 1 Institute of Food Research, Norwich, NR4 7UA, United Kingdom; 2 BioTechnology Institute, University of Minnesota, St. Paul, Minnesota 55108, United States of America; 3 Centre for Infection Medicine, Institute of Microbiology and Epizootics, Freie Universität Berlin, 14163 Berlin, Germany; 4 Department of Molecular Biology and Biotechnology, University of Sheffield, Sheffield, S10 2TN, United Kingdom; University of Louisville, UNITED STATES

## Abstract

The metabolism of *S*. Typhimurium within infected host cells plays a fundamental role in virulence since it enables intracellular proliferation and dissemination and affects the innate immune response. An essential requirement for the intracellular replication of *S*. Typhimurium is the need to regenerate ATP. The metabolic route used to fulfil this requirement is the subject of the present study. For infection models we used human and murine epithelial and macrophage cell lines. The epithelial cell lines were mIC_c12_, a transimmortalised murine colon enterocyte cell line that shows many of the characteristics of a primary epithelial cell line, and HeLa cells. The model macrophage cell lines were THP-1A human monocyte/macrophages and RAW 264.7 murine macrophages. Using a mutational approach combined with an exometabolomic analysis, we showed that neither fermentative metabolism nor anaerobic respiration play major roles in energy generation in any of the cell lines studied. Rather, we identified overflow metabolism to acetate and lactate as the foremost route by which *S*. Typhimurium fulfils its energy requirements.

## Introduction

*Salmonella* is an enteric pathogen responsible for a variety of disease outcomes in humans and animals ranging from self-limited gastroenteritis to lethal typhoid fever. It is estimated that worldwide, typhoidal and non-typhoidal *Salmonella* infections result in an estimated 20 and 98.3 million human cases each year, of which 200,000 and 155,000 result in death respectively [1, 2]. During the infection process, *Salmonella* invades epithelial cells lining the small intestine, mediated by *Salmonella* Pathogenicity Island 1 (SPI1) which encodes a type 3 secretion system (T3SS) which injects effector proteins into the host cell to facilitate uptake of bacteria (invasion) [3]. Intracellular *Salmonella* deploy a second T3SS encoded within SPI2, which modifies the initial membrane-bound compartment or phagosome to form the ‘*Salmonella* containing vacuole’ (SCV) [4]. The SCV is resistant to fusion with lysosomes, enabling *Salmonella* to avoid antimicrobial compounds. In systemic infections, *Salmonella* passes through the gut wall and is phagocytosed by macrophages which can carry the pathogen to systemic sites within the host. Evidence suggests that SPI2 may also be involved in dissemination of *S*. Typhimurium within certain organs, at least within the murine infection model [5].

One of the most intriguing and highly relevant questions regarding *Salmonella* infection is the metabolic adaptations required to enable intracellular replication of *Salmonella* bacteria within host cells, and how these contribute to the overall pathogenicity of the organism. The study of the metabolic requirements for intracellular replication of *S*. Typhimurium within host cells has mainly relied on mutational analysis, isotopologue labelling and proteomic analyses [6–12]. Key insights have arisen from all of these techniques. Previous mutational approaches in a murine carcinoma macrophage cell line have demonstrated that glycolysis and glucose are both essential for intracellular replication and survival of *S*. Typhimurium, and yet several TCA cycle enzymes do not appear to be essential [7, 8]. The latter results are somewhat in contrast to studies in HeLa epithelial cells which showed that deletion of the same TCA cycle genes resulted in an attenuated phenotype in HeLa cells and that glycolysis and glucose were not essential for intracellular replication [6]. The differences in nutritional and metabolic requirements between macrophages and HeLa cells prompted us to investigate whether this was also the case for a more physiologically and metabolically appropriate epithelial cell line. We chose the mIC_c12_ cell line, which in contrast to cancerous epithelial cell lines such as HeLa, is a mouse small intestine enterocyte cell line transimmortalised with SV40 [13]. The mIC_c12_ cells have many of the characteristics of primary enterocyte cells and form a confluent monolayer of cuboid cells separated by tight junctions; they develop dense, short microvilli and form domes, exhibit polarisation and retain the differentiated functions of intestinal crypt cells. In response to infection, they produce a range of chemokines including MCP-1, MIP-2 MIP-1α and β [13]. The mIC_c12_ cell line has been used previously in to determine the antimicrobial effects of secreted chemokines on *Salmonella* [14]. In this study, we use a mutational approach coupled with exometabolite analysis to identify the metabolic routes by which *S*. Typhimurium generates the ATP required for replication within mIC_c12_ epithelial and THP-1A macrophage cell lines. We also contrast the results with similar experiments performed on murine (RAW 264.7) macrophages and HeLa cells. Firstly, we confirm the strict requirement for glycolysis for replication of *S*. Typhimurium in all cell lines, except HeLa cells, and that glucose is a major, but not the only substrate for *S*. Typhimurium in all host cell lines apart from THP-1A macrophages. Our further results then suggest that, in mIC_c12_ and HeLa epithelial cells, *S*. Typhimurium can fulfil its ATP requirements via substrate level phosphorylation (SLP) and/or oxidative phosphorylation (oxphos), however fermentative metabolism and anaerobic respiration play relatively minor roles in intracellular replication. Instead, the data suggests that overflow metabolism and oxphos can satisfy the energetic requirements of *S*. Typhimurium for replication within these epithelial cell lines. However, in the macrophage cell lines studied, *S*. Typhimurium metabolism appears to be further restricted and the electron transport chain (ETC) may be inactive, thus restricting the role of oxphos in ATP generation.

## Results

### Glycolysis is essential for replication of *S*. Typhimurium within mIC_c12_ and THP-1A macrophages but not HeLa’s

**Fig 1:**
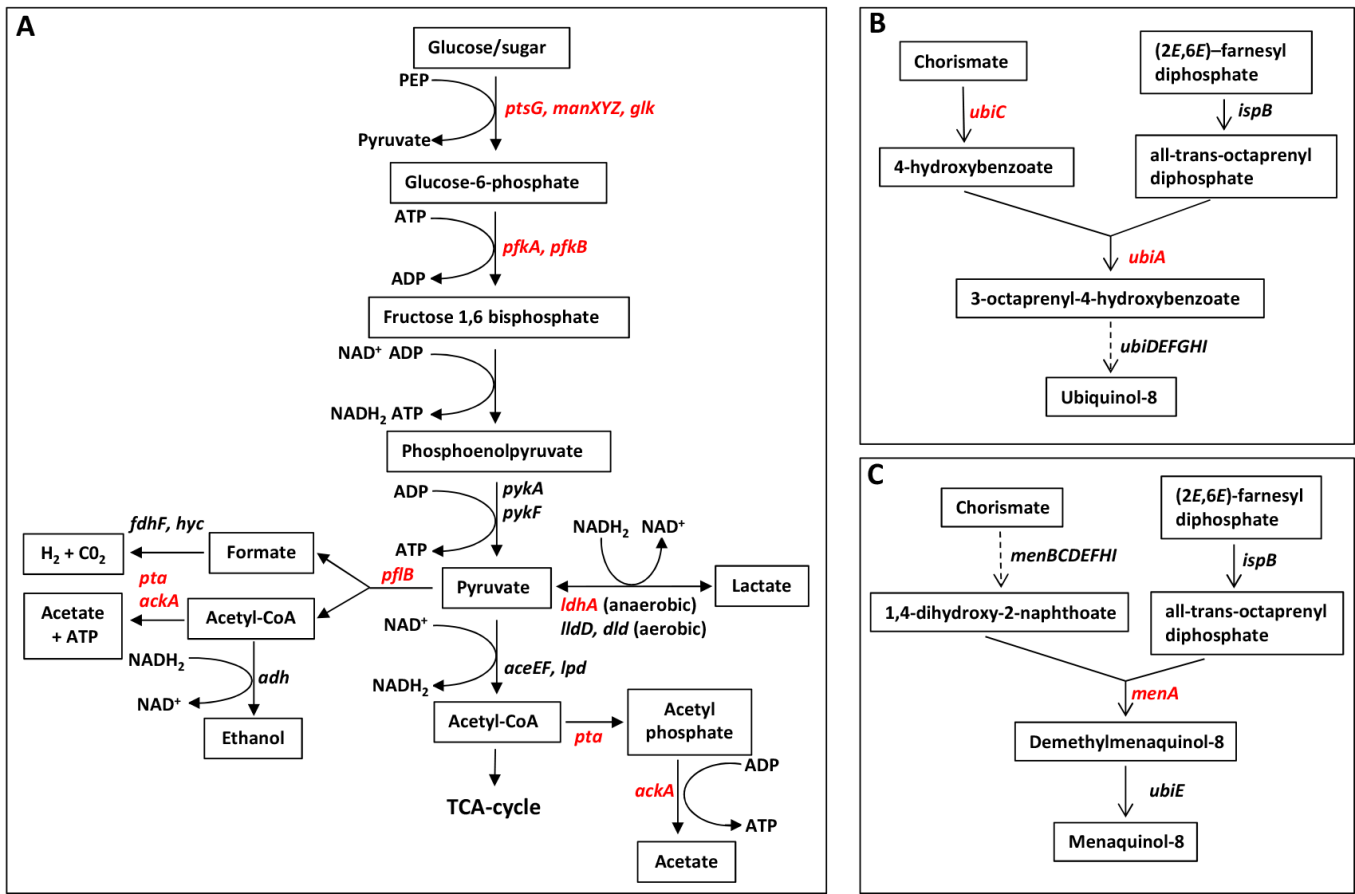
Outline of glycolysis and mixed acid fermentation (A) and ubiquinone (B) and menaquinone biosynthesis (C) with deleted genes shown in red font. The *menA* gene encodes 1,4-dihydroxy-2-naphthoate octaprenyltransferase, *ubiC* = chorismate lyase, *ubiA* = 4-hydroxybenzoate octaprenyltransferase.

Glycolysis is one of the three major sugar catabolic pathways found within bacteria that convert sugars into pyruvate with the concomitant synthesis of ATP and NADH. The enzyme phosphofructokinase is a key committing step in glycolysis and irreversibly converts β-D-fructose 6-phosphate into β-D-fructose1,6-bisphosphate; loss of phosphofructokinase completely blocks glycolysis (Fig 1A). Phosphofructokinase is encoded by two genes in most bacteria designated *pfkA* and *pfkB*. In *Escherichia coli* there are two isozymes of phosphofructokinase (Pfk-1 and Pfk-2). Pfk-1 is a homotetrameric enzyme and the subunits are encoded by *pfkA*. Pfk-2 is a homodimer and the subunits are encoded by *pfkB*. Less than 5% of the Pfk activity in *E*. *coli* can be attributed to Pfk-2 [15].

**Fig 2:**
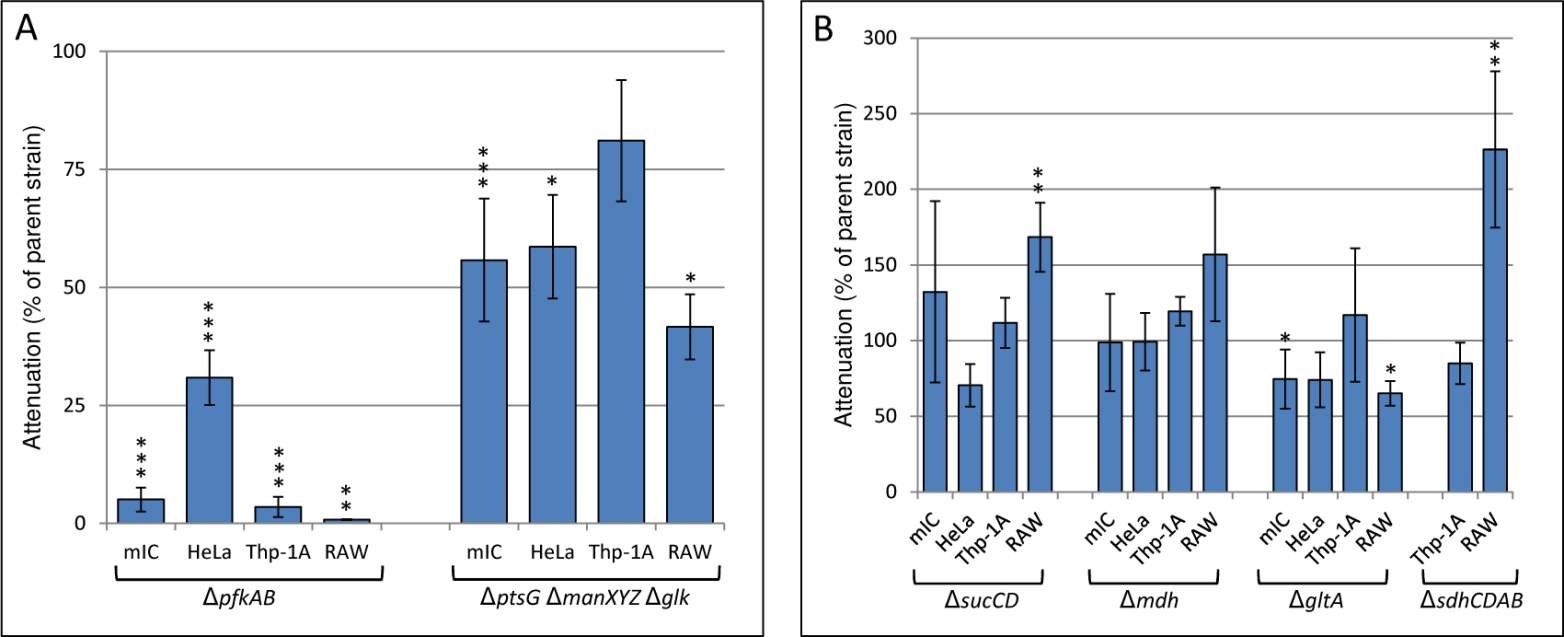
Infection assays of *S*. Typhimurium 4/74 parental and mutant strains in epithelial cells and macrophages. The charts show the percentage attenuation in mIC_c12_, HeLa, THP-1A and RAW 264.7 cells for the following mutant strains relative to the parent strain. (A) Δ*pfkAB*, Δ*ptsG*Δ*manXYZ*Δ*glk*. (B) Δ*sucCD*, Δ*mdh*, Δ*gltA*, *ΔsucCD*, Δ*sdhCDAB* (mIC_c12_, HeLa infections not determined for latter strain). Error bars represent the standard deviation from at least three independent biological replicates performed on separate days and significant differences between parental strain 4/74 and the mutant strains are indicated by asterisks, as follows: no asterisk, *P* > 0.05; *, *P* < 0.05; **, *P* < 0.01; and ***, *P* < 0.001. Replicate data and statistical analysis is from S2 Table.

We tested an *S*. Typhimurium Δ*pfkAB* mutant for its ability to invade and replicate within mIC_c12_ cells. As shown in Fig 2A, we found that the strain was unable to replicate within mIC_c12_ cells suggesting that glycolysis is essential for the replication of *S*. Typhimurium within these cells (complementation data for replication in all cell lines is shown in S2 Fig). Work by others on the requirement for glycolysis in infected host cells has shown that *eno*, *fba*, *pgk*, *gapA* and *tpiA* deficient strains of *S*. Typhimurium are strongly attenuated in RAW 264.7 macrophages [16]. A strict requirement for phosphofructokinase was also found for replication of *S*. Typhimurium within THP-1A (and RAW 264.7) macrophage cell lines as has previously been demonstrated [8], (Fig 2A). The latter results were in contrast to HeLa cells where the intracellular replication rate of the Δ*pfkAB* strain was reduced by 70% compared to the parental strain, suggesting glycolysis is slightly less important for replication of *S*. Typhimurium in this cell line [6]. It should be noted that it is possible that potential accumulation of toxic phosphorylated glycolytic intermediates such as glucose-6-phosphate [17] may have some effect on reducing replication of the *S*. Typhimurium Δ*pfkAB* strain, however, no severe growth defects were observed in *in vitro* grown cultures of the latter strain relative to the parent in minimal media containing glucose as sole carbon source (data not shown). In addition, we also observed attenuated phenotypes of the *S*. Typhimurium glucose transport mutant (Δ*ptsG*Δ*manXYZ*Δ*glk*) in certain host cell lines (Fig 2A and described in the following section), suggesting glucose is an important substrate, and most likely catabolised via the glycolytic pathway.

### Requirement for glucose for efficient replication of *S*. Typhimurium within mIC_c12_ cells, HeLa’s and THP-1A macrophages

The importance of glycolysis for replication of *S*. Typhimurium within the macrophage and epithelial cell lines used in this study suggested hexose sugar(s) were a major catabolic substrate. Of the potential carbohydrates entering via the glycolytic pathway, previous and current work indicated that glucose was required for replication of *S*. Typhimurium in RAW 264.7 macrophages (Fig 2A and [8]). This has been shown using an *S*. Typhimurium Δ*ptsG*Δ*manXYZ*Δ*glk* strain, which is unable to transport glucose [8], and where replication was reduced to 42% of the parent strain in RAW 264.7 macrophages (Fig 2A, S2 Table). In contrast, there was no significant difference in the replication of the Δ*ptsG*Δ*manXYZ*Δ*glk* strain compared to the parent strain in THP-1A macrophages, showing glucose is not an important substrate in this cell line (Fig 2A, S2 Table). However the strong replication defect of the Δ*pfkAB* strain in THP-1A macrophages suggests an alternative glycolytic carbohydrate is important. In the mIC_c12_ and HeLa cell lines, replication of the Δ*ptsG*Δ*manXYZ*Δ*glk* strain was reduced by 44% and 41% respectively compared to the parent strain. The latter observation indicates that although glucose is required for efficient replication within these cell lines, it is not an essential substrate (Fig 2A, S2 Table); again, the high attenuation of the Δ*pfkAB* strain suggests alternate glycolytic substrates are available within these cell lines.

### An intact TCA cycle is not required for efficient replication of *S*. Typhimurium within mIC_c12_ epithelial cells or THP-1A macrophages

Having previously demonstrated that an intact TCA cycle was not required for replication of RAW 264.7 macrophages [7], we decided to test whether this was also the case for *S*. Typhimurium within THP-1A macrophages and mIC_c12_ and HeLa epithelial cells. We used strains harbouring deletions of genes encoding the following enzymes involved in the TCA cycle: succinyl-CoA synthetase (Δ*sucCD*), malate dehydrogenase (Δ*mdh*), citrate synthase (Δ*gltA*) and succinate dehydrogenase (Δ*sdhCDAB*). As described previously, we found that the Δ*sucCD*, Δ*mdh* and Δ*sdhCDAB* strains showed increased recovery from infected RAW 264.7 macrophages compared to the parent strain [7], (Fig 2B). For THP-1A macrophages, we found that the *S*. Typhimurium Δ*gltA*, Δ*sucCD*, Δ*mdh* and Δ*sdhCDAB* strains showed no significant differences in intracellular cfu’s compared to the parent strain after 18h infection (Fig 2B, S2 Table). For infected mIC_c12_ cells, we also found that there was no significant difference in recovered cfu’s of the Δ*sucCD* and Δ*mdh* strains compared to the parent strain and a slight reduction (25%) of the Δ*gltA* strain. For infected HeLa cells, there was a reduction in recovered cfu’s of the Δ*sucCD* and Δ*gltA* strains compare to the parent strain, although this was not significant (Fig 2B, S2 Table). Together, the results demonstrate that *S*. Typhimurium does not require an intact TCA cycle for efficient replication in any of the host cell lines studied. The latter observation could imply that oxphos may not be completely necessary for intracellular replication and that biosynthetic intermediates derived from the TCA cycle such as amino acids might be available from other sources (e.g. host cells, tissue culture media).

### ATP synthase is essential for replication of *S*. Typhimurium within THP-1A and RAW 267.4 macrophages but not mIC_c12_ or HeLa epithelial cell lines

**Fig 3:**
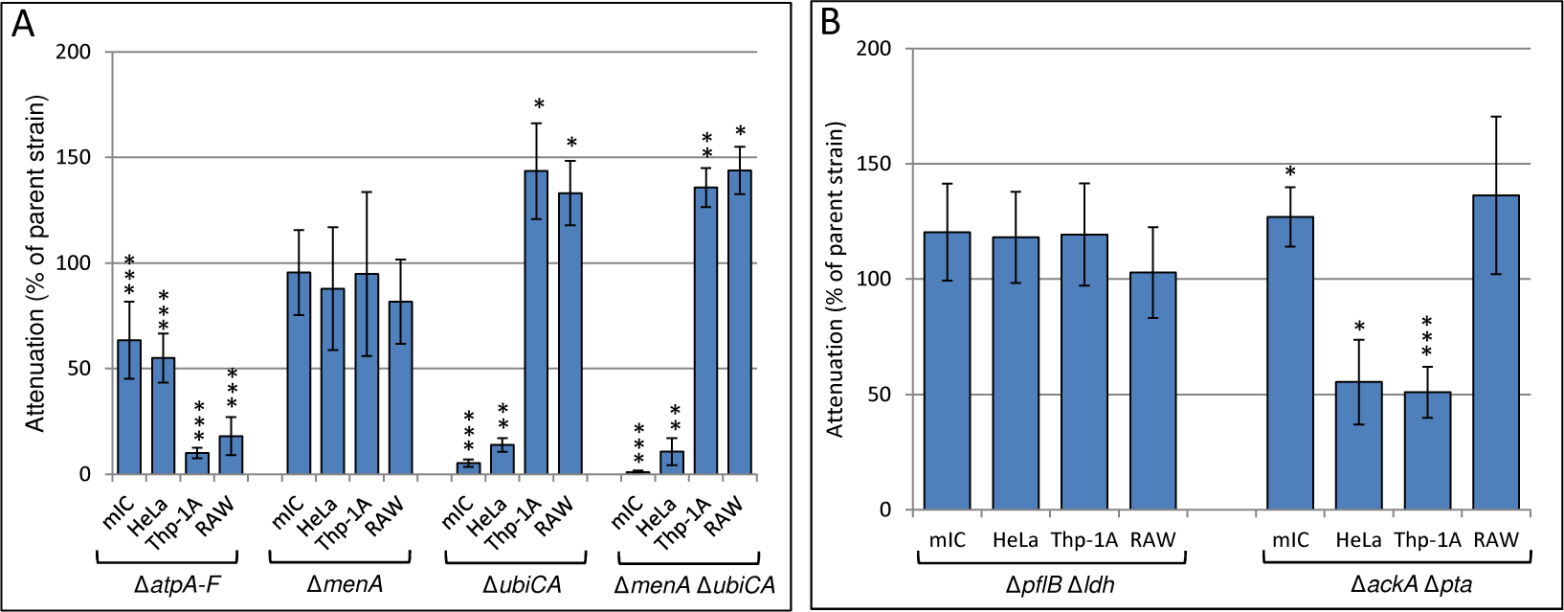
Infection assays of *S*. Typhimurium 4/74 parental and mutant strains in epithelial cells and macrophages. The charts show the percentage attenuation in mIC_c12_, HeLa, THP-1A and RAW 264.7 host cells for the following mutant strains relative to the parent strain. (A) Δ*atpA-F*, Δ*menA*, Δ*ubiCA*, Δ*menAΔubiCA* (B) Δ*pflB*Δ*ldhA*, Δ*ackA*Δ*pta* Error bars represent the standard deviation from at least three independent biological replicates performed on separate days and significant differences between parental strain 4/74 and the mutant strains are indicated by asterisks, as follows: no asterisk, *P* > 0.05; *, *P* < 0.05; **, *P* < 0.01; and ***, *P* < 0.001. Replicate data and statistical analysis is from S2 Table.

The requirement of glycolysis for efficient replication of *S*. Typhimurium within the macrophage and the epithelial cell lines used in this study, and the lack of requirement for an intact TCA cycle (Fig 2A and 2B) led us to consider whether oxidative phosphorylation (oxphos) or substrate level phosphorylation (SLP), or potentially both could supply intracellular *Salmonella* with the majority of its ATP requirements [18]. Substrate level phosphorylation can occur during glycolysis by the conversion of 1,3-bisphospho-D-glycerate to 3-phospho-D-glycerate by phosphoglycerate kinase and from the conversion of phosphoenolpyruvate to pyruvate by pyruvate kinase (Fig 1A). Substrate level phosphorylation also generates ATP in the TCA cycle via the conversion of succinyl-CoA to succinate by succinyl-CoA synthetase and also potentially during fermentation via the conversion of acetyl phosphate to acetate by acetate kinase (Fig 1A), [19]. If oxphos was essential for replication within the host cell lines considered in this study, then ATP synthase would also be required. We therefore deleted the entire ATP synthase operon (Δ*atpI-C*) and tested the ability of the latter mutant to replicate within mIC_c12_, HeLa, THP-1A and RAW 264.7 cells. Fig 3A shows that replication of the Δ*atpI-C* strain was significantly reduced to 63% and 55% respectively of the parent strain within mIC_c12_ and HeLa cells, showing that although ATP synthase contributes to the replication of *S*. Typhimurium within these cell lines it is not essential. In contrast, replication of the *S*. Typhimurium Δ*atpI-C* strain in THP-1A and RAW 264.7 macrophages was strongly reduced to 10% and 18% respectively relative to the parent strain (Fig 3A, S2 Table). Complementation data is shown in S2 Fig.

### Ubiquinone is required for replication of *S*. Typhimurium within mIC_c12_ and HeLa cells but not THP-1A or RAW 264.7 macrophages

The reduced requirement for ATP synthase for efficient replication of *S*. Typhimurium within the mIC_c12_ and HeLa epithelial cell lines compared to the THP-1A and RAW 264.7 macrophage cell lines led us to consider the necessity for an active electron transport chain (ETC) for replication of *S*. Typhimurium within these cell lines. Ubiquinone and menaquinone are key components of the ETC embedded within the bacterial cytoplasmic membranes where they function as hydrogen carriers. Ubiquinone and menaquinone are essential for aerobic and anaerobic respiration respectively. We constructed *S*. Typhimurium strains containing deletions of either the ubiquinone (*ubiCA*) or menaquinone (*menA*) genes (or both; Δ*ubiCA*Δ*menA* strain). These genes encode key enzymes involved in the biosynthetic pathways of ubiquinone and menaquinone (Fig 1B and 1C), and it has been shown previously that deletion of the *ubiCA* and *menA* genes disrupts synthesis of ubiquinone or menaquinone respectively [20, 21]. The mutant strains were tested for replication within HeLa and mIC_c12_ epithelial cells as well as THP-1A and RAW 264.7 macrophages. The data shows that there was no significant difference in replication of the Δ*menA* strain compared to the parent strain in any of the host cell lines tested (Fig 3A, S2 Table). Interestingly, recovery of the Δ*ubiCA* or Δ*ubiCA*Δ*menA* strains from both macrophage cell lines was significantly increased relative to the parent strain; however, in contrast, the Δ*ubiCA* or Δ*ubiCA*Δ*menA* strains were highly attenuated within HeLa and mIC_c12_ epithelial cells (Fig 3A).

### Fermentation is not necessary for the efficient replication of *S*. Typhimurium within RAW 264.7 and THP-1A macrophages and mIC_c12_ and HeLa cells

The partial attenuation of the Δ*atpI-C* strain in mIC_cl2_ and HeLa cells, and the lack of requirement for ubiquinone or menaquinone for replication of *S*. Typhimurium in the macrophage cell lines might suggest that fermentation is supplying intracellular *S*. Typhimurium with a proportion of its ATP requirements.

In order to test this hypothesis, we deleted the genes encoding pyruvate formate-lyase and the anaerobically induced lactate dehydrogenase (*pflB* and *ldhA* respectively), (Fig 1A). Pyruvate formate-lyase anaerobically converts pyruvate to formate and acetyl-CoA and the fermentative D-lactate dehydrogenase converts pyruvate to lactate under anaerobic conditions at acidic pH [22]. An *E*. *coli* strain in which the *pflB* and *ldhA* genes have been deleted is unable to ferment due to its inability to achieve redox balance during growth [22]. Similarly, we showed that an *S*. Typhimurium Δ*pflB*Δ*ldhA* double mutant was unable to grow *in vitro* under fermentative conditions (S1 Fig). When we tested the *S*. Typhimurium Δ*pflB*Δ*ldhA* strain for its ability to replicate in the epithelial and macrophage cell lines used in this study, we found that there was no significant difference compared to the parent strain for any of the cell lines (Fig 3B), implying that fermentative metabolism is not necessary for intracellular replication.

### Exometabolite production by *S*. Typhimurium within macrophages and epithelial cell lines

**Fig 4:**
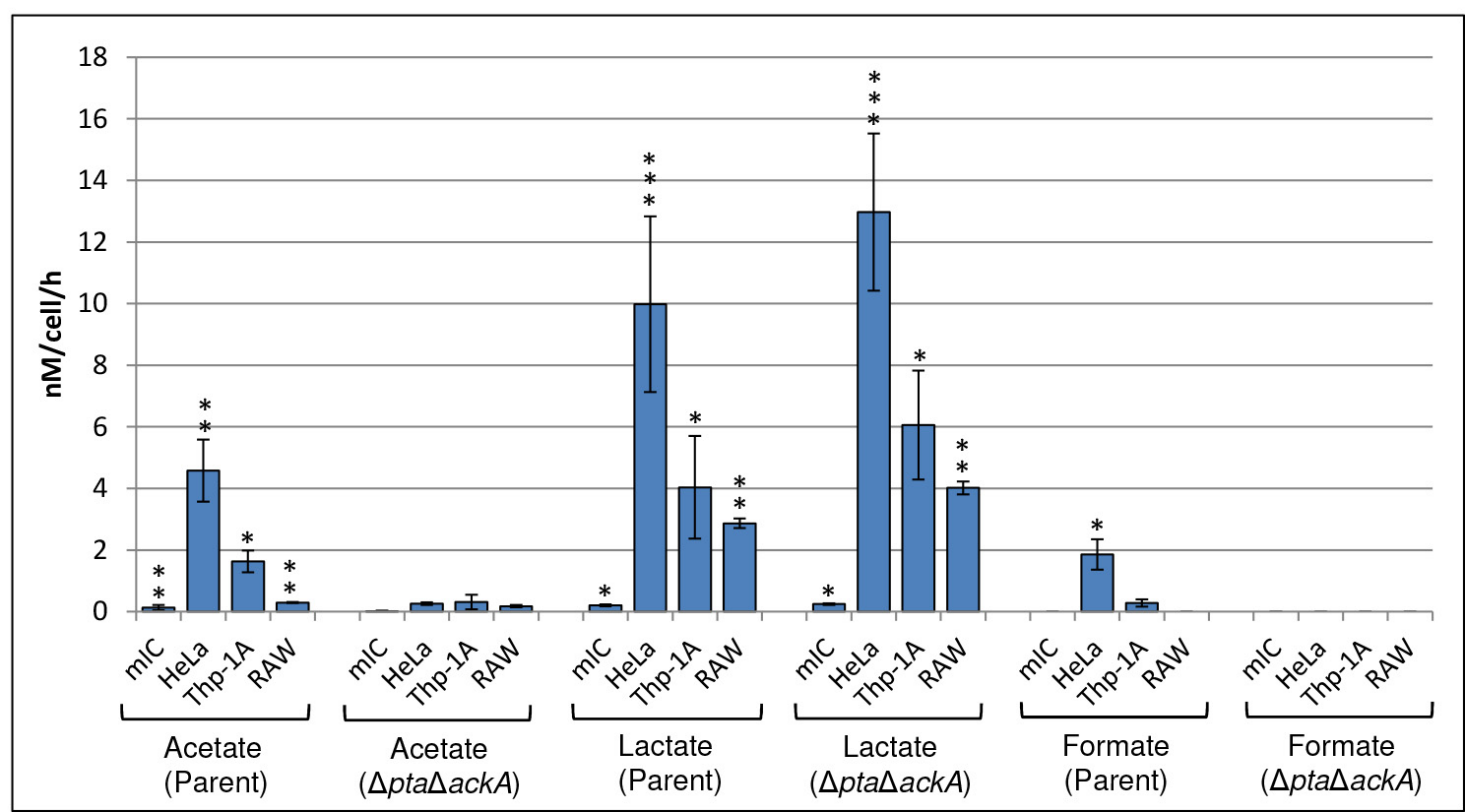
Exometabolite concentrations of acetate, lactate and formate produced by *S*. Typhimurium within macrophages and epithelial cells. Concentrations of acetate lactate and formate are shown for all host cell lines for the *S*. Typhimurium parent strain (4/74) or Δ*pta*Δ*ackA* strain as indicated. The data was corrected for exometabolite production by uninfected host cells. Error bars represent the standard deviation from at least three independent biological replicates performed on separate days and significant differences between infected and uninfected medium is indicated by asterisks, as follows: no asterisk, *P* > 0.05; *, *P* < 0.05; **, *P* < 0.01; and ***, *P* < 0.001. The data is presented as average concentrations of exometabolite produced per bacterial cell, per hour.

The results described above suggested that fermentative metabolism played a minor role, if any, in the replication of *S*. Typhimurium in the host cell lines used in this study. In order to underpin this finding and to potentially obtain further insight into the intracellular metabolism of *S*. Typhimurium we used 1D ^1^H-NMR of cell culture media to monitor excretion of metabolites (exometabolites) produced during infection of host cells. The NMR spectra were analysed for acetate, succinate, fumarate, ethanol, lactate and formate (after controlling for potential production of these metabolites by uninfected host cells and cell culture medium, and also by host cells infected with heat inactivated bacteria). We were unable to detect significant quantities of succinate, fumarate, ethanol or formate excreted from infected mIC_c12_ cells or THP-1A or RAW 264.7 macrophages, and only a relatively small quantity of formate from infected HeLa cells (Fig 4). However significant concentrations of lactate and acetate were found compared to uninfected host cells for all of the host cell lines studied (Fig 4). The highest concentrations of both lactate and acetate were found to be excreted from infected HeLa cells, followed by THP-1A and RAW 264.7 macrophage cell lines, and the lowest, but significant concentrations were excreted by infected mIC_c12_ cells (Fig 4). That excreted acetate was not being produced by host cells as a result of infection was demonstrated using an *S*. Typhimurium Δ*pta*Δ*ackA* strain in which the conversion of acetyl-CoA to acetate was unable to occur; we were unable to detect significant quantities of acetate excreted by host cells infected with the latter strain (Fig 4). In addition to acetate, we also monitored lactate and formate production in the Δ*pta*Δ*ackA* strain. We were unable to detect formate in post-infection cell culture medium from any of the cell lines infected with the Δ*pta*Δ*ackA* strain, however, lactate production was increased slightly by 1.2–1.5 fold), but this was not significant (Fig 4). Decreased formate and elevated lactate production in an *E*.*coli* Δ*pta* mutant has previously been noted [23].

Both acetate and lactate can be produced as a result of ‘overflow metabolism’ or the ‘bacterial Crabtree effect’ [24–26]. Overflow metabolism can occur under aerobic conditions and at relatively high glucose levels where the carbon flux from acetyl-CoA is mainly directed to acetate and lactate instead of, or in addition to entering the TCA cycle (Fig 1A). Under such conditions the role of oxidative phosphorylation is reduced and the high glycolytic flux generates the majority of ATP (via SLP) for growth. The aerobic production of lactate can serve to re-oxidise some of the NADH produced during glycolysis, and has also been shown to regenerate the transmembrane proton gradient, and therefore ATP [27]. Two enzymes are involved in the conversion of acetyl-CoA to acetate: phosphotransacetylase and acetate kinase [28]. The former enzyme, (encoded by the *pta* gene), results in the synthesis of acetyl phosphate which is then converted to acetate by acetate kinase (encoded by the *ackA* gene) with the concomitant production of ATP (Fig 1A). In order to determine the possible contribution of the latter pathway in replication of *S*. Typhimurium within host cells, we constructed a Δ*pta*Δ*ackA* deletion strain and tested it in infection assays; Fig 3B shows that replication of the Δ*pta*Δ*ackA* strain was attenuated by ~50% in HeLa and THP-1A cells compared to the parent strain; however in mIC_c12_ cells replication was slightly increased and in RAW 264.7 macrophages there was no difference compared to the parent strain.

## Discussion

In this study, we performed a mutational and exometabolomic analysis to determine the requirement for central metabolic pathways related to ATP generation that enable the replication of *S*. Typhimurium within mIC_c12_ and HeLa epithelial cells and THP-1A and RAW 267.4 macrophage cells. Key mutants in genes involved in glycolysis, the TCA cycle, fermentation, oxidative phosphorylation, and electron transfer were used to gain an insight into ATP generation by intracellular *S*. Typhimurium. We determined intracellular replication of the mutant strains within all host cell lines at two time points during infection, a final time point of 18h for the macrophage lines (and mIC_c12_ cells), and 9h for HeLa cells, and also at an intermediate time point (S3 Fig). For all of the mutant strains in all of the host cell lines we found the same trends, in terms of percentage replication relative to the parent strain, at the intermediate and final time points (S3 Fig).

Our analysis showed firstly that glycolysis is required for efficient replication of *S*. Typhimurium in HeLa and mIC_c12_ cells and within THP-1A and RAW 264.7 macrophages. The latter observation supports previously published data showing the requirement of glycolysis for replication of *S*. Typhimurium within RAW 264.7 macrophages [8, 16]. In HeLa cells, glycolysis was found not to be essential for replication of *S*. Typhimurium, also as previously shown [6]. Although glucose is a glycolytic substrate and present in tissue culture medium, we found that it was differentially required by *Salmonella* within the cell lines studied here; for example, in THP-1A macrophages, lack of glucose transport/uptake had no significant effect on the final level of replication of *S*. Typhimurium (Fig 2A). However, it is noted that the concentrations of glucose present in tissue culture medium may be a contributory factor to the occurrence of overflow metabolism in tissue culture based infection assays. However, intracellular glucose concentrations in other epithelial cell lines and THP-1A macrophages have been estimated at 100–200 μM (K. Tedin, pers. comm.) which is well within the estimated range potentially occurring in human and murine intestinal epithelial cells *in vivo* [29–31].

The requirement for glycolysis led us to consider whether ATP synthase and therefore oxphos were required for replication of *S*. Typhimurium within host cell lines. Our data indicated that ATP synthase was not absolutely essential for replication of *S*. Typhimurium within mIC_c12_ and HeLa epithelial cell lines; the *S*. Typhimurium Δ*atpI-C* strain was attenuated by 37% and 45% respectively in these host cell lines relative to the parent strain. This result suggested that oxphos and/or SLP can provide the ATP requirements necessary for replication of *Salmonella* within these cell lines; this metabolic flexibility is also suggested by the lack of requirement for an intact TCA cycle for replication of *S*. Typhimurium (Fig 2B). In support of intracellular *Salmonella* potentially using oxphos as a means of ATP generation, the requirement for an active ETC was demonstrated by the necessity for ubiquinone for replication of *S*. Typhimurium within mIC_c12_ and HeLa cells. However, in the case of HeLa cells, the production of considerable quantities of excreted lactate and acetate, and a relatively small amount of formate from intracellular *Salmonella* may suggest SLP can also provide sufficient ATP for some replication. In infected mIC_c12_ cells, the much reduced concentrations of lactate and acetate produced relative to infected HeLa cells could suggest oxphos is a major route of ATP generation. However, in the absence of ATP synthase, intracellular *Salmonella* may be sufficiently metabolically flexible to produce enough ATP via SLP for replication in mIC_c12_ cells (the metabolic flexibility of intracellular *Salmonella* has been highlighted elsewhere [32, 33]). The presence of considerable cytosolic subpopulations of *S*. Typhimurium within HeLa cells has recently been demonstrated [34, 35]; such subpopulations may also occupy a distinct metabolic niche compared to intravacuolar *Salmonella* which may also explain the partial attenuation of the ATP synthase mutant within these host cell lines. For both mIC_c12_ and HeLa cells, the lack of requirement for menaquinone suggests anaerobic respiration is not required for replication of *Salmonella*.

In distinct contrast to the epithelial cell lines, the *S*. Typhimurium Δ*atpI-C* strain was highly attenuated in the macrophage cell lines, which may have suggested oxphos plays a major role in ATP generation. Surprisingly however, neither ubiquinone nor menaquinone biosynthesis was required for replication of *S*. Typhimurium within macrophages. Indeed, significantly higher cfu’s from the Δ*ubiCA* and Δ*ubiCA*Δ*menA* strains were recovered compared to the parent strains (Fig 3A). The latter result appears to support the prior demonstration that the ETC is inactive in macrophages due to the effect of the respiratory oxidative burst [36], and perhaps the reason for the apparent ‘hyper-replication’ of the Δ*ubiCA* and Δ*ubiCA*Δ*menA* strains may be due to reduction of further oxidative stress in the latter strains relative to the parent. Despite the lack of requirement for ubiquinone or menaquinone for replication of *S*. Typhimurium in macrophages, one explanation for the high attenuation of the Δ*atpI-C* strain in macrophages may be that ATP synthase is acting in reverse as a proton pump ATPase to reduce the detrimental effects of acidification caused by the defensive macrophage v-ATPase. This could also potentially reduce the effects of acidification caused by the lactate and acetate produced by intracellular *Salmonella* in macrophages (Fig 4, [37, 38]). Indeed, the function of ATP synthase acting ‘in reverse’ as a proton pump is a well-documented mechanism for reducing acidification caused by organic acids in fermenting bacteria [39–42].

In contrast to the infected macrophage cell lines, the Δ*ubiCA* and Δ*ubiCA*Δ*menA* strains were highly attenuated within HeLa and mIC_c12_ epithelial cells (Fig 3A). The latter results indicate that oxphos and/or hydrogen transport are necessary for efficient growth of *S*. Typhimurium within epithelial cells but not macrophages; however the partial attenuation of the Δ*atpI-C* strain in epithelial cells suggests ATP synthesis (via SLP) can to some extent substitute for loss of ATP synthetase. The stringent requirement for ubiquinone in the infected epithelial cell lines may suggest respiration, and generation of a transmembrane proton gradient is essential for processes in addition to ATP synthesis (e.g. solute transport). Finally, with respect to the apparent lack of strict requirement for oxphos in intracellular S. Typhimurium, it is perhaps of relevance that recent findings have elucidated a link between ATP and virulence in *Salmonella*. it has been shown that a virulence protein (MgtC) interacts with the *a* subunit of the F_1_F_o_ ATP synthase, hindering ATP-driven proton translocation and NADH-driven ATP synthesis in inverted vesicles, and that high levels of intracellular ATP can cause decreased virulence and result in growth arrest in low magnesium media [43–45].

Withn all of the host cell lines used in this study, the lack of attenuation of the *S*. Typhimurium Δ*pflB*Δ*ldhA* strain demonstrates that redox control via fermentative metabolism is not a significant metabolic requirement for replication of *Salmonella*. The production of a relatively small quantity of formate in HeLa cells (Fig 4) may suggest some fermentation does occur in a subpopulation of *S*. Typhimurium in HeLa cells, but the absence of fermentation had no significant impact on replication. The above observations, together with the lack of attenuation of the Δ*menA* mutant, suggests that the host cell intracellular environment is mostly aerobic, as has previously been suggested [46]. The exometabolic production of significant quantities acetate and lactate by *S*. Typhimurium within all of the host cell lines studied (Fig 4) suggests that glycolytic overflow is the principal route which enables the replication of *Salmonella* within the epithelial and macrophage cell lines used in this study. The latter phenomenon, well known in commercial production strains of *E*. *coli* where high glucose or sugar concentrations are used, can occur aerobically under conditions of high glycolytic flux [24–27]. Instead of, or in addition to entering the TCA cycle, the acetyl-CoA produced is diverted to acetate and/or lactate; the production of lactate is well known to occur under aerobic conditions due to the action of L-lactate dehydrogenase (LldD) [47, 48], (Fig 1A). The production of acetate can provide a further growth advantage due to the synthesis of ATP from the conversion of acetyl phosphate to acetate by acetate kinase, and the production of lactate helps to regenerate the NAD reduced during glycolysis, ([27], Fig 1A). It should be noted that overflow metabolism does not preclude oxphos as a means of NADH oxidation and ATP generation. The production of ATP by acetate kinase may provide a possible reason for attenuation of the Δ*pta*Δ*ackA* strain in HeLa cells and THP-1A macrophages compared to mIC_c12_ cells and RAW 264.7 macrophages (Fig 3B). In corroboration of the latter observation it was found that *S*. Typhimurium within HeLa and THP-1A cells produced significantly higher levels of excreted acetate compared to infected mIC_c12_ and RAW 264.7 macrophages (Fig 4). In terms of virulence determinants, the production of quantities of organic acids in intracellular *Salmonella* may impact SPI1 expression since it has been shown that acetate induces SPI1 expression [49–51], and lactate reduces *hilA* expression [52]. The latter results suggest that glycolytic flux could impinge on the temporal dynamics of the *Salmonella* virulence regulons that in turn may account for host cell specific infection cycles.

## Methods

### Bacterial strains, growth conditions and reagents

*S*. Typhimurium strains and plasmids used in this work are listed in S1 Table. Strains were maintained in Luria-Bertani (LB) broth or on plates with appropriate antibiotics at the following concentrations; ampicillin (Sigma Aldrich), 100 μg.ml^-1^; chloramphenicol (Cm, Sigma Aldrich), 12.5 μg.ml^-1^; kanamycin (Kn, Sigma Aldrich), 50 μg.ml^-1^; tetracycline (Tet, Sigma Aldrich), 15 μg.ml^-1^. M9 minimal medium with 0.4% w/v glucose was used where indicated. Oligonucleotide primers were purchased from Sigma Genosys or Illumina.

### Mutant strain construction

*S*. Typhimurium mutant strains were constructed according to published procedures [17] and as briefly described in [12]. Transductants were screened on green agar plates to obtain lysogen-free colonies [18]. The complete absence of the structural genes was verified by DNA sequencing of the deleted regions of the chromosome. The FLP-recombinase encoded on pCP20 was used to remove the antibiotic resistance markers as described in [17].

### Epithelial cell infection assays

Infection assays in human HeLa epithelial cells (obtained from American Type Culture Collection, Rockville, MD) were performed according to [16]. Briefly, HeLa cells were grown in DMEM medium (Sigma, D5546) containing 1 g.L^-1^ glucose and supplemented with 10% fetal bovine serum (Sigma), 2mM L-glutamine (Sigma) and 20mM HEPES buffer (Sigma). Between 1 and 3 x10^5^ HeLa cells were seeded into each well of a 6- or 12-well cell culture plate and infected with *S*. Typhimurium 4/74 and mutant strains at an MOI of 10:1. Prior to infection the *S*. Typhimurium strains had been grown to an OD_600_ of 1.2 to allow expression of the SPI1 Type 3 secretion system.

To increase the uptake of *Salmonella*, plates were centrifuged at 1000 g for 5 min, and this was defined as time 0 h. After 1 h of infection, extracellular bacteria were killed with 30 μg.ml^-1^ gentamicin. The media was replaced after 1 h with medium containing 5 μg.ml^-1^ gentamicin. Incubations were continued for 2 h and 6 h. To estimate the amount of intracellular bacteria at each time point, cells were lysed using 0.1% SDS, and samples were taken for viable counts [19]. Statistical significances were assessed by using Student’s unpaired *t*-test, and a *P* value of <0.05 was considered significant.

The infection procedure for mIC_c12_ cells (obtained from Prof. S. Carding, IFR, Norwich), was essentially the same, except the medium used was DMEM-F12 (sigma D6434); 5 μg.ml^-1^ insulin (sigma I1882); 50 nM dexamethasone (sigma D8893); 60 nM sodium selenite (sigma S9133); 5 μg.ml^-1^ transferrin (sigma T1428); 1 nM triiodothyronine (sigma T5516); 10 ng ml^-1^ EGF from mouse (sigma E4127); 20 mM Hepes (sigma H0887); 2 mM L-glutamine (sigma G7513); 2% Fetal Bovine Serum (sigma F7524); 1 g.L^-1^ D-glucose (sigma G8644). Infection was carried out at an MOI of 1 and allowed to proceed for 2h and 18h. The recovered cfu’s at 18h relative to 2h was used as an estimate of intracellular growth. Statistical significances were assessed by using Student’s unpaired *t*-test, and a *P* value of <0.05 was considered significant (S2 Table).

### Macrophage infection assays

Infection assays in murine RAW 264.7 macrophages (obtained from American Type Culture Collection; Rockville, MD; ATCC# TIB-71) were performed essentially as previously described [27]. Macrophages were cultured in were grown in MEM medium (Sigma, M0268) containing 1 g.L^-1^ glucose and supplemented with 10% fetal bovine serum (Sigma), 2mM L-glutamine (Sigma) and 20mM HEPES buffer (Sigma). The multiplicity of infection (MOI) for all experiments was 10:1. The infection assays were allowed to proceed for 2 h and 18 h post infection. To estimate the amount of intracellular bacteria at each time point, cells were lysed using 1% Triton X-100 (Sigma), and samples were taken for viable counts [10]. Statistical significance was assessed by using Student’s unpaired *t* test, and a *P* value of 0.05 was considered significant (S2 Table). Infection assays in THP-1A macrophages (an adherent derivative of the THP-1 cell line as described in [51]) were performed as described above except the medium used was IMDM (Sigma I3390), 10% Fetal Bovine Serum (Sigma F7524), 4 mM L-glutamine (Sigma G7513).

### NMR analysis

^1^H Nuclear Magnetic Resonance (^1^H NMR) was used to identify the presence, absence, and concentration of several metabolites in growth medium. The spent growth medium samples were thawed at room temperature and prepared for ^1^H NMR spectroscopy by mixing 400 μL of spent medium with 200 μL of a solution made up in 100% D_2_O containing 0.12% w/v sodium azide, and 1.6mM TSP (sodium 3-(trimethylsilyl)-propionated_4_) as a chemical shift reference. The sample was mixed, and 500 μL was transferred into a 5-mm NMR tube for spectral acquisition. The ^1^H NMR spectra were recorded at 600MHz on a Bruker Avance spectrometer (Bruker BioSpin GmbH, Rheinstetten, Germany) running Topspin 2.0 software and fitted with a cryoprobe and a 60-slot autosampler. Each ^1^H NMR spectrum was acquired with 64 scans, a spectral width of 12295 Hz, an acquisition time of 2.67 s, and a relaxation delay of 3.0 s. The “noesypr1d” presaturation sequence was used to suppress the residual water signal with a low-power selective irradiation at the water frequency during the recycle delay and a mixing time of 10 ms. Spectra were transformed with a 0.3-Hz line broadening, manually phased, baseline corrected, and referenced by setting the TSP methyl signal to 0 ppm. Absolute concentrations were obtained by using CHENOMX software (version 5.1) with quantification calculated relative to TSP.

## Supporting Information

S1 Fig Growth phenotypes of 4/74 parental strain and Δ*ldhA*, Δ*pflB* and Δ*ldhA*Δ*pflB* strains in LB under fermentative conditions (docx file). (DOCX)

S2 Fig Complementation of *S*. Typhimurium Δ*pfkAB* and Δ*atpC-I* mutants within all host cell lines. (DOCX)

S3 Fig Replication of *S*. Typhimurium metabolic mutants relative to the parent strain within host cells at intermediate time points. (DOCX)

S1 Table Strains and plasmids used in this study (docx file). (DOCX)

S2 Table Data used for statistical analysis for Figs 2 and 3 (xlsx file). (XLSX)

## References

[1] Chimalizeni Y, Kawaza K, Molyneux E. The epidemiology and management of non typhoidal *Salmonella* infections. Advances in experimental medicine and biology. 2010;659:33–46. 10.1007/978-1-4419-0981-7_3 . 20204753

[2] Pui CF, Wong W.C., Chai L.C., Tunung R., Jeyalectchumi P., Noor Hidayah M.S., Ubong A., Farinazleen M.G., Cheah Y.K., Son R. *Salmonella*: A foodborne pathogen. International Food Research Journal. 2011;18(3):465–73.

[3] Que F, Wu S, Huang R. *Salmonella* pathogenicity island 1 (SPI-1) at work. Current microbiology. 2013;66(6):582–7. 10.1007/s00284-013-0307-8 . 23370732

[4] Haraga A, Ohlson MB, Miller SI. *Salmonella*e interplay with host cells. Nature reviews. 2008;6(1):53–66. . 18026123 10.1038/nrmicro1788

[5] Grant AJ, Morgan FJ, McKinley TJ, Foster GL, Maskell DJ, Mastroeni P. Attenuated *Salmonella* Typhimurium lacking the pathogenicity island-2 type 3 secretion system grow to high bacterial numbers inside phagocytes in mice. PLoS pathogens. 2012;8(12):e1003070 10.1371/journal.ppat.1003070 23236281 PMC3516571

[6] Bowden SD, Hopper-Chidlaw AC, Rice CJ, Ramachandran VK, Kelly DJ, Thompson A. Nutritional and Metabolic Requirements for the Infection of HeLa Cells by *Salmonella enterica* serovar Typhimurium. PloS one. 2014;9(5):e96266 10.1371/journal.pone.0096266 24797930 PMC4010460

[7] Bowden SD, Ramachandran VK, Knudsen GM, Hinton JC, Thompson A. An incomplete TCA cycle increases survival of *Salmonella* Typhimurium during infection of resting and activated murine macrophages. PloS one. 2010;5(11):e13871 10.1371/journal.pone.0013871 21079785 PMC2975626

[8] Bowden SD, Rowley G, Hinton JC, Thompson A. Glucose and glycolysis are required for the successful infection of macrophages and mice by *Salmonella enterica* serovar Typhimurium. Infection and immunity. 2009;77(7):3117–26. 10.1128/IAI.00093-09 19380470 PMC2708584

[9] Gotz A, Eylert E, Eisenreich W, Goebel W. Carbon metabolism of enterobacterial human pathogens growing in epithelial colorectal adenocarcinoma (Caco-2) cells. PloS one. 2010;5(5):e10586 10.1371/journal.pone.0010586 20485672 PMC2868055

[10] Eisenreich W, Dandekar T, Heesemann J, Goebel W. Carbon metabolism of intracellular bacterial pathogens and possible links to virulence. Nature reviews. 2010;8(6):401–12. 10.1038/nrmicro2351 . 20453875

[11] Shi L, Chowdhury SM, Smallwood HS, Yoon H, Mottaz-Brewer HM, Norbeck AD, et al Proteomic investigation of the time course responses of RAW 264.7 macrophages to infection with *Salmonella enterica*. Infection and immunity. 2009;77(8):3227–33. 10.1128/IAI.00063-09 19528222 PMC2715674

[12] Steeb B, Claudi B, Burton NA, Tienz P, Schmidt A, Farhan H, et al Parallel exploitation of diverse host nutrients enhances *Salmonella* virulence. PLoS pathogens. 2013;9(4):e1003301 10.1371/journal.ppat.1003301 23633950 PMC3636032

[13] Bens M, Bogdanova A, Cluzeaud F, Miquerol L, Kerneis S, Kraehenbuhl JP, et al Transimmortalized mouse intestinal cells (m-IC_c12_) that maintain a crypt phenotype. The American journal of physiology. 1996;270(6 Pt 1):C1666–74. . 8764149 10.1152/ajpcell.1996.270.6.C1666

[14] Kotarsky K, Sitnik KM, Stenstad H, Kotarsky H, Schmidtchen A, Koslowski M, et al A novel role for constitutively expressed epithelial-derived chemokines as antibacterial peptides in the intestinal mucosa. Mucosal Immunol. 2010;3(1):40–8. 10.1038/mi.2009.115 . 19812544

[15] Kotlarz D, Garreau H, Buc H. Regulation of the amount and of the activity of phosphofructokinases and pyruvate kinases in *Escherichia coli*. Biochimica et biophysica acta. 1975;381(2):257–68. . 122902 10.1016/0304-4165(75)90232-9

[16] Dandekar T, Astrid F, Jasmin P, Hensel M. *Salmonella enterica*: a surprisingly well-adapted intracellular lifestyle. Front Microbiol. 2012;3:164 10.3389/fmicb.2012.00164 22563326 PMC3342586

[17] Morita T, El-Kazzaz W, Tanaka Y, Inada T, Aiba H. Accumulation of glucose 6-phosphate or fructose 6-phosphate is responsible for destabilization of glucose transporter mRNA in *Escherichia coli*. The Journal of biological chemistry. 2003;278(18):15608–14. 10.1074/jbc.M300177200 . 12578824

[18] Fraenkel DG. Glycolysis. 2 ed. Neidhardt FC, editor. Washington DC: ASM Press; 1996. 189–98 p.

[19] Bock A, Sawers G. Fermentation Neidhardt et al, editor. Washington DC.: ASM Press; 1996. 262–82 p.

[20] Meganathan R. Ubiquinone biosynthesis in microorganisms. FEMS microbiology letters. 2001;203(2):131–9. . 11583838 10.1111/j.1574-6968.2001.tb10831.x

[21] Meganathan R, Kwon O. Biosynthesis of Menaquinone (Vitamin K) and Ubiquinone (Coenzyme Q). Ecosal Plus. 2009;3 10.1128/ecosalplus.3.6.3.3 PMC4172378 26443765

[22] Mat-Jan F, Alam KY, Clark DP. Mutants of *Escherichia coli* deficient in the fermentative lactate dehydrogenase. Journal of bacteriology. 1989;171(1):342–8. 2644194 10.1128/jb.171.1.342-348.1989 PMC209593

[23] Yang YT, Bennett GN, San KY. Effect of inactivation of *nuo* and *ackA-pta* on redistribution of metabolic fluxes in *Escherichia coli*. Biotechnol Bioeng. 1999;65(3):291–7. . 10486127

[24] Paczia N, Nilgen A, Lehmann T, Gatgens J, Wiechert W, Noack S. Extensive exometabolome analysis reveals extended overflow metabolism in various microorganisms. Microb Cell Fact. 2012;11:122 10.1186/1475-2859-11-122 22963408 PMC3526501

[25] Valgepea K, Adamberg K, Nahku R, Lahtvee PJ, Arike L, Vilu R. Systems biology approach reveals that overflow metabolism of acetate in *Escherichia coli* is triggered by carbon catabolite repression of acetyl-CoA synthetase. BMC Syst Biol. 2010;4:166 10.1186/1752-0509-4-166 21122111 PMC3014970

[26] Vemuri GN, Altman E, Sangurdekar DP, Khodursky AB, Eiteman MA. Overflow metabolism in *Escherichia coli* during steady-state growth: transcriptional regulation and effect of the redox ratio. Applied and environmental microbiology. 2006;72(5):3653–61. 10.1128/AEM.72.5.3653-3661.2006 16672514 PMC1472329

[27] El-Mansi M. Flux to acetate and lactate excretions in industrial fermentations: physiological and biochemical implications. J Ind Microbiol Biotechnol. 2004;31(7):295–300. 10.1007/s10295-004-0149-2 . 15257440

[28] Keseler IM, Mackie A, Peralta-Gil M, Santos-Zavaleta A, Gama-Castro S, Bonavides-Martinez C, et al EcoCyc: fusing model organism databases with systems biology. Nucleic acids research. 2013;41(Database issue):D605–12. 10.1093/nar/gks1027 23143106 PMC3531154

[29] Wright EM, Loo DD, Hirayama BA. Biology of human sodium glucose transporters. Physiol Rev. 2011;91(2):733–94. 10.1152/physrev.00055.2009 . 21527736

[30] Stumpel F, Burcelin R, Jungermann K, Thorens B. Normal kinetics of intestinal glucose absorption in the absence of GLUT2: evidence for a transport pathway requiring glucose phosphorylation and transfer into the endoplasmic reticulum. Proceedings of the National Academy of Sciences of the United States of America. 2001;98(20):11330–5. 10.1073/pnas.211357698 11562503 PMC58729

[31] Naftalin RJ. Does apical membrane GLUT2 have a role in intestinal glucose uptake? F1000Res. 2014;3:304 10.12688/f1000research.5934.1 ; PubMed Central PMCID: PMCPMC4309173. 25671087 PMC4309173

[32] Barat S, Steeb B, Maze A, Bumann D. Extensive in vivo resilience of persistent *Salmonella*. PloS one. 2012;7(7):e42007 10.1371/journal.pone.0042007 22911873 PMC3404010

[33] Becker D, Selbach M, Rollenhagen C, Ballmaier M, Meyer TF, Mann M, et al Robust *Salmonella* metabolism limits possibilities for new antimicrobials. Nature. 2006;440(7082):303–7. . 16541065 10.1038/nature04616

[34] Knodler LA, Nair V, Steele-Mortimer O. Quantitative assessment of cytosolic *Salmonella* in epithelial cells. PloS one. 2014;9(1):e84681 10.1371/journal.pone.0084681 24400108 PMC3882239

[35] Malik-Kale P, Winfree S, Steele-Mortimer O. The bimodal lifestyle of intracellular *Salmonella* in epithelial cells: replication in the cytosol obscures defects in vacuolar replication. PloS one. 2012;7(6):e38732 10.1371/journal.pone.0038732 22719929 PMC3374820

[36] Husain M, Bourret TJ, McCollister BD, Jones-Carson J, Laughlin J, Vazquez-Torres A. Nitric oxide evokes an adaptive response to oxidative stress by arresting respiration. The Journal of biological chemistry. 2008;283(12):7682–9. 10.1074/jbc.M708845200 18198179

[37] Grinstein S, Nanda A, Lukacs G, Rotstein O. V-ATPases in phagocytic cells. J Exp Biol. 1992;172:179–92. . 1491224 10.1242/jeb.172.1.179

[38] Lukacs GL, Rotstein OD, Grinstein S. Phagosomal acidification is mediated by a vacuolar-type H(+)-ATPase in murine macrophages. The Journal of biological chemistry. 1990;265(34):21099–107. . 2147429

[39] Trchounian A. *Escherichia coli* proton-translocating F_0_F_1_-ATP synthase and its association with solute secondary transporters and/or enzymes of anaerobic oxidation-reduction under fermentation. Biochem Biophys Res Commun. 2004;315(4):1051–7. 10.1016/j.bbrc.2004.02.005 . 14985119

[40] Bender GR, Marquis RE. Membrane ATPases and acid tolerance of *Actinomyces viscosus* and *Lactobacillus casei*. Applied and environmental microbiology. 1987;53(9):2124–8. 2445289 10.1128/aem.53.9.2124-2128.1987 PMC204068

[41] Bender GR, Sutton SV, Marquis RE. Acid tolerance, proton permeabilities, and membrane ATPases of oral *streptococci*. Infection and immunity. 1986;53(2):331–8. 3015800 10.1128/iai.53.2.331-338.1986 PMC260879

[42] Quivey RG Jr., Kuhnert WL, Hahn K. Adaptation of oral streptococci to low pH. Adv Microb Physiol. 2000;42:239–74. . 10907552 10.1016/s0065-2911(00)42004-7

[43] Lee EJ, Pontes MH, Groisman EA. A bacterial virulence protein promotes pathogenicity by inhibiting the bacterium's own F_1_F_o_ ATP synthase. Cell. 2013;154(1):146–56. 10.1016/j.cell.2013.06.004 23827679 PMC3736803

[44] Lee EJ, Groisman EA. Control of a *Salmonella* virulence locus by an ATP-sensing leader messenger RNA. Nature. 2012;486(7402):271–5. 10.1038/nature11090 22699622 PMC3711680

[45] Pontes MH, Sevostyanova A, Groisman EA. When Too Much ATP Is Bad for Protein Synthesis. Journal of molecular biology. 2015;427(16):2586–94. 10.1016/j.jmb.2015.06.021 26150063 PMC4531837

[46] Eriksson S, Lucchini S, Thompson A, Rhen M, Hinton JC. Unravelling the biology of macrophage infection by gene expression profiling of intracellular *Salmonella enterica*. Molecular microbiology. 2003;47(1):103–18. . 12492857 10.1046/j.1365-2958.2003.03313.x

[47] Dong JM, Taylor JS, Latour DJ, Iuchi S, Lin EC. Three overlapping lct genes involved in L-lactate utilization by *Escherichia coli*. Journal of bacteriology. 1993;175(20):6671–8. 8407843 10.1128/jb.175.20.6671-6678.1993 PMC206779

[48] Futai M, Kimura H. Inducible membrane-bound L-lactate dehydrogenase from *Escherichia coli*. Purification and properties. The Journal of biological chemistry. 1977;252(16):5820–7. . 18473

[49] Lawhon SD, Maurer R, Suyemoto M, Altier C. Intestinal short-chain fatty acids alter *Salmonella* Typhimurium invasion gene expression and virulence through BarA/SirA. Molecular microbiology. 2002;46(5):1451–64. . 12453229 10.1046/j.1365-2958.2002.03268.x

[50] Rishi P, Pathak S, Ricke SC. Short chain fatty acids influence virulence properties of *Salmonella enterica* serovar Typhimurium. J Environ Sci Health B. 2005;40(4):645–57. 10.1081/PFC-200061576 . 16047886

[51] Van Immerseel F, De Buck J, Pasmans F, Velge P, Bottreau E, Fievez V, et al Invasion of *Salmonella enteritidis* in avian intestinal epithelial cells in vitro is influenced by short-chain fatty acids. International journal of food microbiology. 2003;85(3):237–48. . 12878382 10.1016/s0168-1605(02)00542-1

[52] Durant JA, Corrier DE, Stanker LH, Ricke SC. Expression of the *hilA Salmonella* Typhimurium gene in a poultry *Salm*. *enteritidis* isolate in response to lactate and nutrients. Journal of applied microbiology. 2000;89(1):63–9. . 10945780 10.1046/j.1365-2672.2000.01089.x

