# Supplementary material for: A Comparison of the ATP Generating Pathways Used by S. Typhimurium to Fuel Replication within Human and Murine Macrophage and Epithelial Cell Lines
Source: PLoS One. 2016 Mar 1;11(3):e0150687. doi: 10.1371/journal.pone.0150687 (PMC4773185; doi:10.1371/journal.pone.0150687)
Supplement: S1 Fig — (DOCX) [file pone.0150687.s001.docx]

**Figure S1.** Growth curves for *S*. Typhimurium 4/74 parent strain (blue), Δ*ldhA* (green), Δ*pflB* (red) and Δ*ldhA*Δ*pflB* (purple) strains under oxygen-limited conditions in LB + 0.2% in filled, stoppered 25-ml Bijou bottles.
