# Supplementary material for: A Comparison of the ATP Generating Pathways Used by S. Typhimurium to Fuel Replication within Human and Murine Macrophage and Epithelial Cell Lines
Source: PLoS One. 2016 Mar 1;11(3):e0150687. doi: 10.1371/journal.pone.0150687 (PMC4773185; doi:10.1371/journal.pone.0150687)
Supplement: S2 Fig — (DOCX) [file pone.0150687.s002.docx]

**
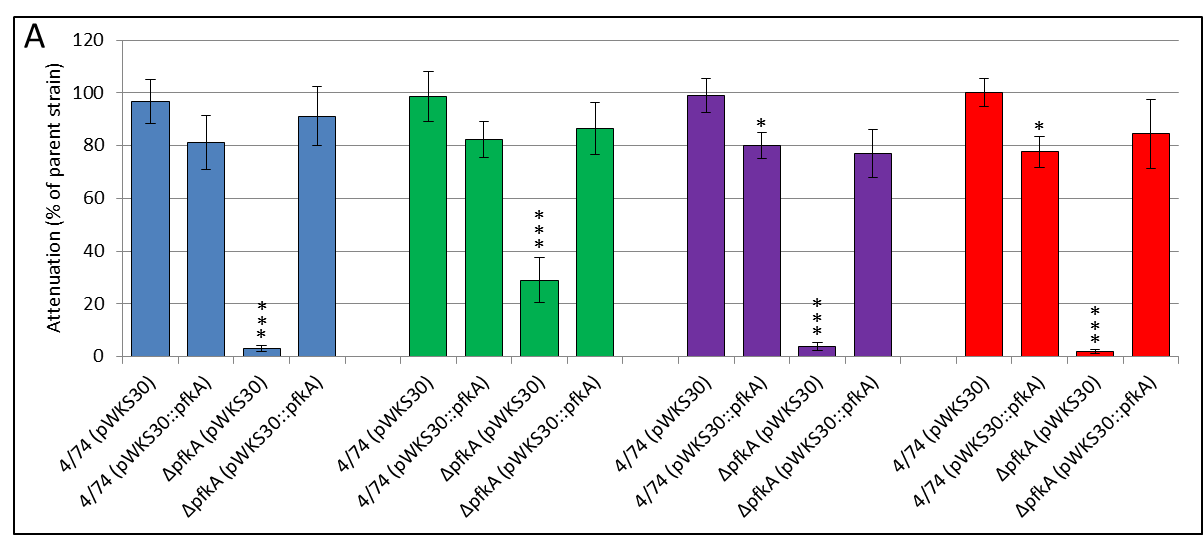
**

**
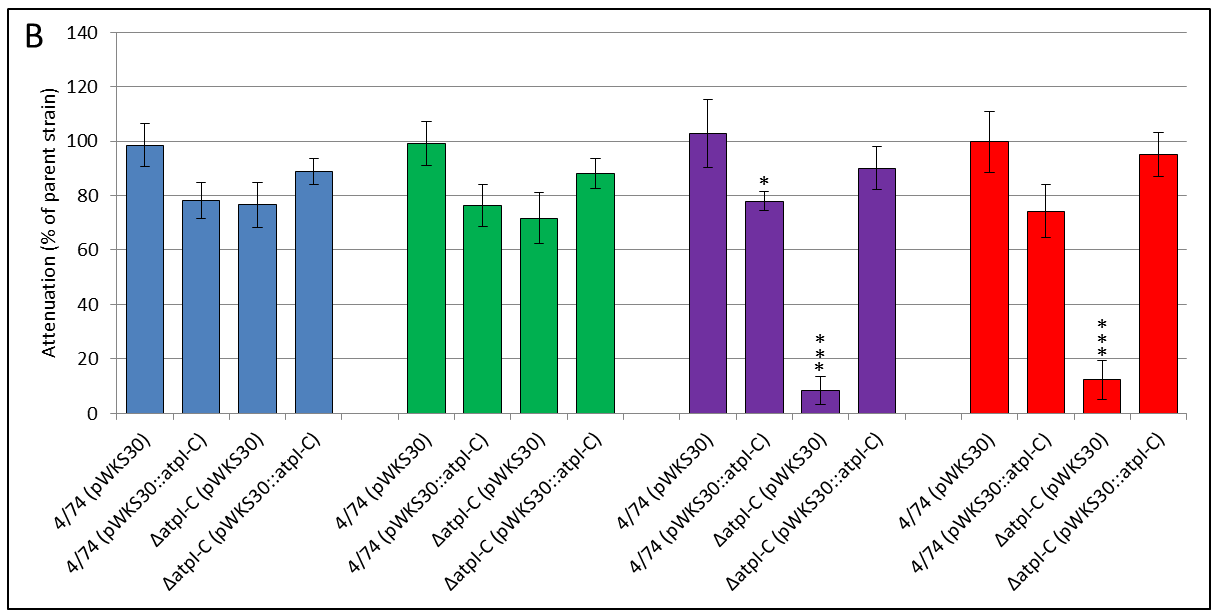
**

**Figure S2.** Complementation of the *S*. Typhimurium *pfkAB and atpI-C* strains in HeLa (blue bars), mIC_c12_ (green bars), THP-1A (purple bars) or RAW 264.7 cell lines (red bars). The data is presented as percentage replication relative to the parent strain without plasmid inside host cell lines at 6 h (HeLa cells) or 18 h post-infection (mIC_c12_, THP-1A and RAW 264.7 cell lines). Error bars represent the standard deviation from at least three independent biological replicates performed on separate days and significant differences between parental strain 4/74 and the mutant or plasmid containing strains are indicated by asterisks, as follows: no asterisk, *P* > 0.05; *****, *P* < 0.05; ******, *P* < 0.01; and *******, *P* < 0.001. Replicate data and statistical analysis is from S2 Table.

**Methods**

Construction of the pWKS30::*pfkA* is as described in [1]. For construction of the pWKS30::*atpC-I* plasmid, the *atpIBEFHAGDC* operon plus 518 bp of upstream sequence was PCR amplified from *S*. Typhimurium 4/74 genomic DNA using primers atpA1 (5_-CGTCTATCTAGACGGTTTCGTTTCAACATGACAACG) and atpA2 (5_-CGTCTAGGGCCCCGCATCCATTCCTCCCTTC). The PCR product was digested with XbaI and ApaI, ligated into the low-copy-number vector pWKS30, and transformed into *Escherichia coli* strain DH5α. The resulting plasmid was designated pWKS30::*atpI-C* and was confirmed by DNA sequencing across the multiple-cloning site using primers M13F (5_-CGCCAGGGTTTTCCCAGTCACGAC) and M13R (5_-TCACACAGGAAACAGCTATGAC). Plasmids pWKS30 and pWKS30::*atpI-C* were then transformed into 4/74 and AT1144 by electroporation.

**References**

1. Bowden S.D, Rowley G., Hinton J.C.D., Thompson, A. (2009) Glucose and glycolysis are required for the successful infection of macrophages and mice by *Salmonella enterica* serovar Typhimurium *Infect. & Immun*. **77**:3117-26.
