# Supplementary material for: A Comparison of the ATP Generating Pathways Used by S. Typhimurium to Fuel Replication within Human and Murine Macrophage and Epithelial Cell Lines
Source: PLoS One. 2016 Mar 1;11(3):e0150687. doi: 10.1371/journal.pone.0150687 (PMC4773185; doi:10.1371/journal.pone.0150687)
Supplement: S3 Fig — (DOCX) [file pone.0150687.s003.docx]

**
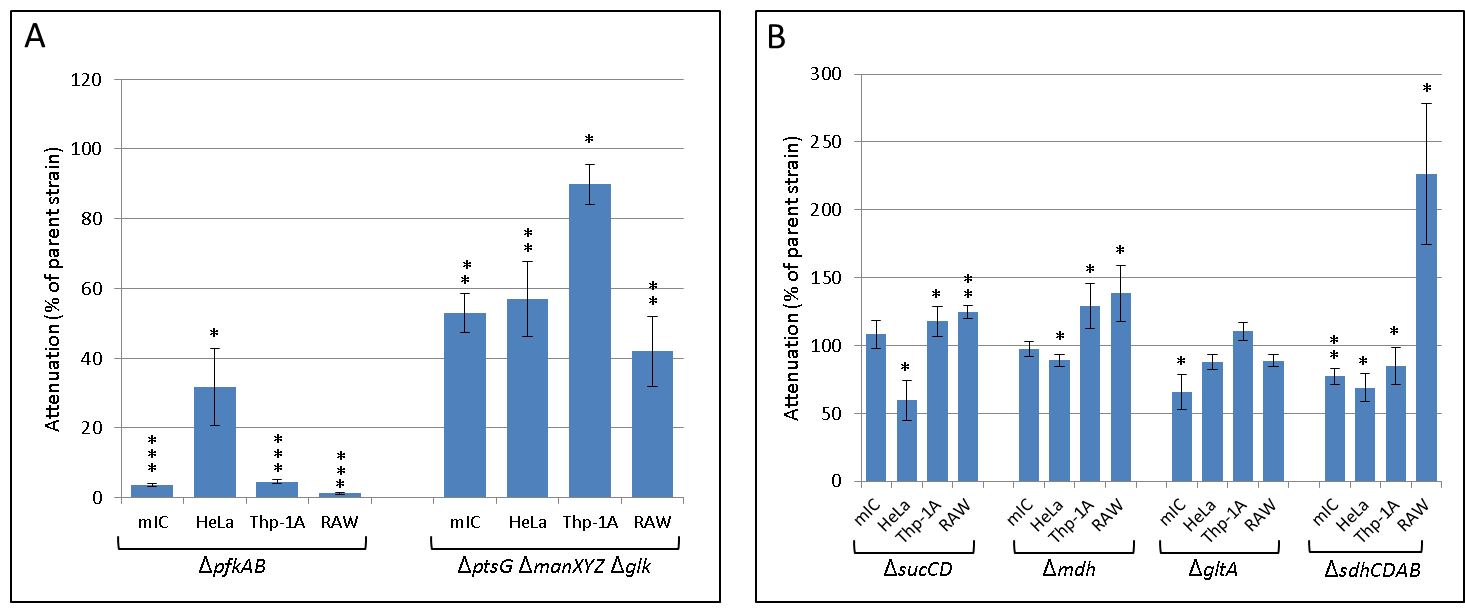
**

**
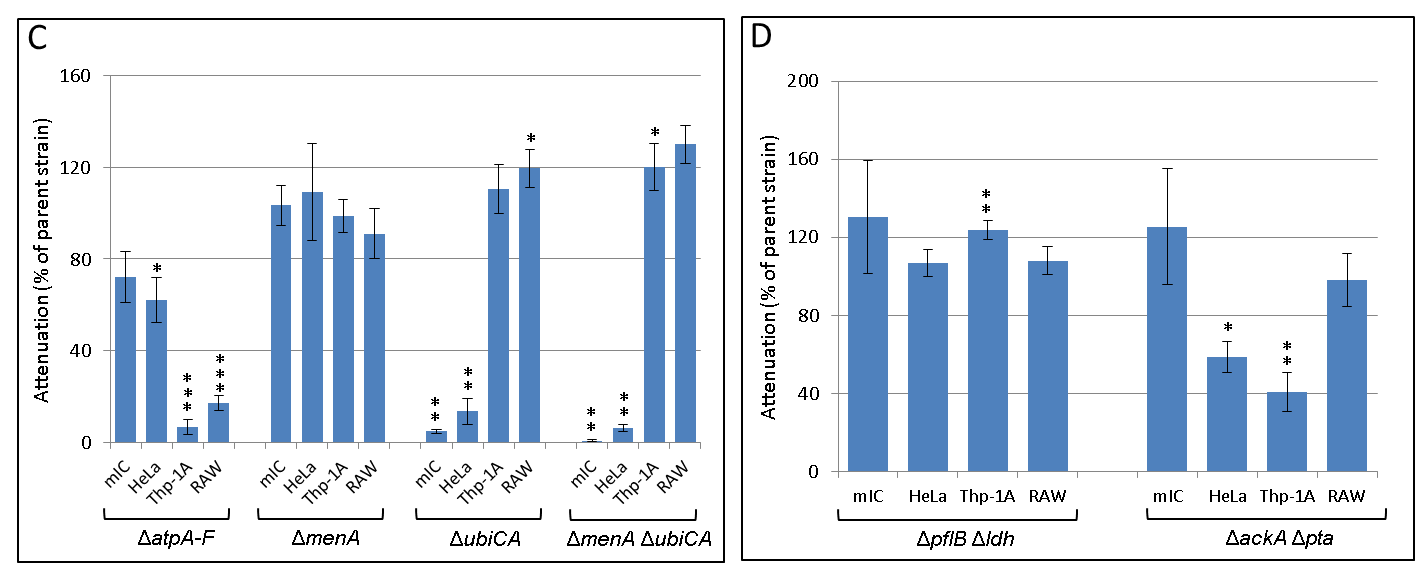
**

**Figure S3.** Replication of *S.* Typhimurium metabolic mutants relative to the parent strain within HeLa cells and macrophages at 2h and 9h post-infection respectively. The charts show the percentage attenuation in mIC_c12_, HeLa, THP-1A and RAW 264.7 cells for the following mutant strains relative to the parent strains. (A) Δ*pfkAB*, Δ*ptsG*Δ*manXYZ*Δ*glk*. (B) Δ*sucCD*, Δ*mdh,* Δ*gltA, ΔsucCD,* Δ*sdhCDAB* (C) Δ*atpA-F*, Δ*menA,* Δ*ubiCA,* Δ*menAΔubiCA* (D) Δ*pflB*Δ*ldhA,* Δ*ackA*Δ*pta* Error bars represent the standard deviation from at least three biological replicates and significant differences between parental strain 4/74 and the mutant strains are indicated by asterisks, as follows: no asterisk, *P* > 0.05; *****, *P* < 0.05; ******, *P* < 0.01; and *******, *P* < 0.001.
