# Supplementary material for: A Comparison of the ATP Generating Pathways Used by S. Typhimurium to Fuel Replication within Human and Murine Macrophage and Epithelial Cell Lines
Source: PLoS One. 2016 Mar 1;11(3):e0150687. doi: 10.1371/journal.pone.0150687 (PMC4773185; doi:10.1371/journal.pone.0150687)
Supplement: S1 Table — (DOCX) [file pone.0150687.s004.docx]

**Table S1** Strains and plasmids used in this study.

| ***S.* Typhimurium strains** | **Relevant genotype** | **Method of construction** | **Reference** |
| --- | --- | --- | --- |
| 4/74 | Parent strain | N/A | [1] |
| JH3486 | 4/74 ∆*pfkA*::Kn ∆*pfkB*::Cm | λ Red mutagenesis | [2] |
| AT1014 | 4/74 Δ*ptsG*::Cm, Δ*manXYZ*, Δ*glk*::Kn | λ Red mutagenesis | [2] |
| JH3449 | 4/74 Δ*sucCD*::Kn | λ Red mutagenesis | [3] |
| JH3505 | 4/74 Δ*gltA*::Kn | λ Red mutagenesis | [3] |
| JH3508 | 4/74 Δ*mdh*::Kn | λ Red mutagenesis | [3] |
| JH3392 | 4/74 Δ*sdhCDAB*::Kn | λ Red mutagenesis | [3] |
| AT1144 | 4/74 Δ*atpA-H*::Kn | λ Red mutagenesis | This study |
| AT1327 | 4/74 Δ*pflB*::Kn Δ*ldh*::Cm | λ Red mutagenesis | This study |
| AT1296 | 4/74 Δ*menA*::Cm | λ Red mutagenesis | This study |
| AT1311 | 4/74 Δ*ubiCA*::Kn | λ Red mutagenesis | This study |
| AT1313 | 4/74 Δ*menA*::Cm Δ*ubiCA*::Kn | λ Red mutagenesis | This study |
| AT1286 | 4/74 Δ*pta* Δ*ackA*::Cm | λ Red mutagenesis | This study |
| **Plasmids** |  |  |  |
| pKD46 | λ Red recombinase expression plasmid | N/A | [4] |
| pKD3 | Cm^R^ resistance cassette-containing plasmid | N/A | [4] |
| pKD4 | Kn^R^ resistance cassette-containing plasmid | N/A | [4] |
| pCP20 | FLP-recombinase expression plasmid | N/A | [4] |

1. Wray C, Sojka WJ (1978) Experimental *Salmonella typhimurium* infection in calves. Res Vet Sci 25: 139-143.
2. Bowden S.D, Rowley G., Hinton J.C.D., Thompson, A. (2009) Glucose and glycolysis are required for the successful infection of macrophages and mice by *Salmonella enterica* serovar Typhimurium *Infect. & Immun*. **77**:3117-26.
3. Bowden S.D, Ramachandran V.K., Knudsen G.M., Hinton J.C., Thompson, A. (2010) An incomplete TCA cycle increases survival of *Salmonella* Typhimurium during infection of resting and activated murine macrophages. *PLoS One*. 5(11):e13871.
4. Datsenko KA, Wanner BL (2000) One-step inactivation of chromosomal genes in *Escherichia coli* K-12 using PCR products. Proc Natl Acad Sci U S A 97: 6640-6645.
